# AskMyDoc: engaging, educating, and empowering global majority communities on health

**DOI:** 10.3389/fpubh.2026.1864483

**Published:** 2026-08-18

**Authors:** Enam Haque, Yasmin Samir

**Affiliations:** 1 Division of Medical Education, School of Medical Sciences, Faculty of Biology, Medicine and Health, Manchester, United Kingdom; 2 AskMyDoc, Manchester, United Kingdom

**Keywords:** AskMyDoc, community engagement, community health awareness, ethnic minority health, global majority, medical education

## Abstract

Health inequities disproportionality affects global majority patients, due to socio-economic disadvantage. This leads to poorer health outcomes, in terms of morbidity and mortality. Community based interventions are ways by which to improve the health of these underserved communities. AskMyDoc is a charity that was founded in 2011, with the aim to engage, educate and empower ethnic minority communities. This paper aims to highlight the work of the organisation, reflecting on the highlights and challenges of breaking down barriers to health for global majority patients. AskMyDoc has engaged through delivery of multiple talks and workshops in local community spaces, providing discussions in multiple languages. It contributed to health literacy during Covid-19, through creation of over 200 videos relating to Covid-19 and cancer screening awareness. Collaboration with charities and health organisations has been key to the success of the work, as has developing medical students and resident doctors through the work. AskMyDoc has demonstrated the positive impact of grassroots health promotion and advocacy. Despite challenges of a sustained volunteer workforce and funding restrictions, it demonstrates a path by which to better serve global majority patients in the United Kingdom.

## Introduction

Health inequities remain a significant public health issue in the UK and globally, particularly affecting people from global majority communities. The term “people of the global majority” has increasingly been adopted by UK public and voluntary organisations in favour of the term “ethnic minorities,” referring to all non-White ethnic groups who together make up most of the world’s population (1). This includes individuals of Black, Asian, Indigenous, Latin, and mixed ethnic backgrounds; earlier terminology such as BAME (Black, Asian and Minority Ethnic) is now used less frequently. Despite their global majority status, these groups often experience poorer health outcomes, reflecting deep-rooted social, economic, and structural inequalities.

The Marmot Review, “Fair Society, Healthy Lives,” was published in 2010 and highlighted how health inequities are shaped by the social determinants of health, including income, education, housing, and access to services (2). People from global majority communities are more likely to face barriers such as socioeconomic disadvantage, discrimination, language barriers, and unequal access to culturally appropriate healthcare in the UK. These factors contribute to disparities in both physical and mental health outcomes and limit opportunities for prevention and early intervention.

There is evidence of differing health outcomes between White and global majority patients in developed countries. Individuals from global majority backgrounds have a higher risk of long-term conditions such as Diabetes and Hypertension (3). Black patients also have higher rates of hypertension and stroke, with earlier average onset of these conditions (3). A systematic review found that South Asians were significantly more likely to die from Cardiovascular Disease than their White counterparts (4). These patterns demonstrate the impact of structural inequalities, emphasising the urgent need for targeted, culturally sensitive interventions to reduce health disparities and improve outcomes within these communities (3).

## Background and rationale

The Marmot Review recommended early intervention and public engagement to address health inequities (5). Looking specifically at global majority communities, the NHS Race & Health Observatory recommended greater investment in these communities, with the advice that the NHS looked at culturally sensible approaches to primary prevention (6). Health inequities can be addressed through community-based interventions. These cost-effective interventions break down barriers to access, and provide a culturally sensitive approach, to maximise engagement with underserved communities in different income level countries (7). An international review of such activities revealed its positive impact on addressing health inequities (8). In the review, they found activities primarily consisting of workshops, covering themes around general health disparities, medical conditions, environmental factors impacting health, and lifestyle measures. There was evidence of co-creation with communities, but unfortunately not involving them in all aspects of each programme. The literature has examples of the success of such interventions. A systematic review looking at the impact of community health workers on colorectal cancer screening noted a significant improvement in patient uptake, compared to patients without this intervention (9). A scoping review looking at community interventions for addressing mental health issues in global majority patients noted four out of the seven studies in the review had demonstrated significant improvement in mental health outcomes, although many of the studies were subject to bias (10).

## Case description

AskMyDoc is a UK charity led by a group of healthcare professionals in Manchester, United Kingdom. The organisation was founded in 2011 by a group of five doctors, with a vision to address health inequalities facing global majority communities living in Manchester. It obtained charity status in 2023, and has a vision to engage, educate and empower global majority patients. AskMyDoc has worked with local community groups in Greater Manchester, as well as national health charities, over the last 15 years to promote health through various community events in different settings. This paper aims to outline their work, as well as reflect on the highlights and challenges of the grassroots work.

### Outreach activities

The bedrock of AskMyDoc’s activities has been talks and workshops to global majority communities in Greater Manchester, in areas of deprivation. Over 15 years, they have delivered over a hundred events, including health awareness workshops on depression, men’s health, women’s health, cardiovascular health, diabetes, stroke, mental health, and dementia. Many of their activities have included collaboration with other charities, such as the British Heart Foundation, Diabetes UK, and the Hepatitis C Trust. A highlight in 2016 was collaborating with Target Ovarian Cancer and other charities, to deliver talks on ovarian and breast cancer awareness in various locations. Another was supporting the Christie Hospital in Manchester in improving global majority patient participation in the innovative NHS Galleri trial, which aimed to detect risks of 50 cancers with a simple blood test. The trial recently came under scrutiny, with the finding that the test did not statistically significantly reduce onset of late-stage cancer (11).

### Greater Manchester Hepatitis C strategy

AskMyDoc was invited by Public Health North-west and the Hepatitis C Trust, onto a project to increase awareness of Hepatitis C in the Pakistani community in Greater Manchester in 2015 and 2016. As well as delivering a talk in a Manchester Mosque, they held a stall at the Manchester Mega Mela. Both events had a bus present, which enabled healthcare volunteers to perform OraSure testing of attendees. Public Health England, in their Hepatitis C in the Northwest Annual report 2016, noted that these events resulted in “engagement with 1,182 individuals and 445 individuals tested at a mosque and the Mega Mela Festival in Manchester over 6 days between 2015 and 2016” (12).

### Media activities

Covid-19 negatively impacted global majority communities more than their White counterparts, partly due to health inequities caused by greater poverty, frontline jobs, and overcrowded housing (13). A paper reviewing health information studies during this time, noted there was little quality information of for those who spoke languages other than English (12). This led to poor knowledge of the condition, widespread misinformation, and distrust of governments and their vaccine programme (14).

The AskMyDoc team noted the potential worsening health inequities for those who did not speak English. They began to develop short informational videos on Covid-19 in different languages, through a dedicated YouTube page and social media platforms. The information was based on government guidance, to ensure the accuracy of the information. This enabled patients who did not speak English, to understand government advice during the height of the pandemic. The work required the recruitment of a team of “AskMyDoc Champions,” a team of healthcare volunteers fluent in different languages. Volunteers responded to a social media request to provide their time to create standardised videos in their native language, based on an English script provided by AskMyDoc. As Covid-19 eased, the team continued with videos. Answer Cancer is a Greater Manchester initiative to improve cancer screening (15). It commissioned them to provide videos on cancer screening. Approximately 30 AskMyDoc Champions helped to produce 280 videos in 24 different languages over a period of 3 years. This work enabled patients to have free access to invaluable resources in different languages.

During Covid-19, there was also a need for engagement with other key members of the community. AskMyDoc invited Imams and community leaders to a series of Covid Vaccine seminars, to highlight key information to them, and to debunk myths. This was with the aim to ensure health literacy from other trusted individuals in the community.

AskMyDoc was recognised as a major contributor to health awareness in global majority communities. In view of this, the team was invited in 2021 to an expert panel on the Covid-19 vaccine, organised by the South Asian Heritage Month team, a webinar with 918 views (16).

Reflecting on this work, AskMyDoc provided a much-needed intervention to support global majority communities. However, it took a great toll on the Trustees involved in the work. They spent many hours reviewing and editing the videos, as well as liaising with different Champions. The video output ended, once the cancer awareness videos were completed, due to the lack of administrative support for this activity. If an organisation wishes to undertake similar work as a sustainable project, we recommend that they employ a dedicated media lead equipped with the time and skills to deliver on this work.

### Empowering learners

An important way to ensure sustainability in community engagement work is to ensure empowerment of learners. AskMyDoc has achieved this through development of medical student working groups. Trustees supervised groups of medical students, to design and deliver workshops on specific health conditions. They also attended the workshops with the students, providing senior support to questions from members of the community. The legacy of this initiative was that two students eventually became Trustees of the charity.

Students have been encouraged to collaborate with AskMyDoc, on projects looking at global majority health. In 2016, a medical student devised an educational session for Pakistani patients with poorly controlled Diabetes, identified by GP surgeries in a Manchester locality. The student used evidence-based approaches to highlight the dangers of poorly controlled Diabetes, and the importance of dietary changes and weight loss, to improve HBA1C levels.

AskMyDoc continues to engage the student body. In 2019, it supported the development of the University of Manchester AskMyDoc Student Society, affiliated with the Students Union. This organisation delivered health awareness sessions for students, including topics such as organ donation and healthcare issues affecting refugees and asylum seekers.

AskMyDoc Trustees supported a medical student in 2021, to deliver online webinars on different health topics to Bangladeshi women. “Amar Doctor” was delivered in Bengali, with a global audience.

The University of Manchester 2035 Strategy includes values-led and socially responsible work such as partner engaged learning (17). In line with this, AskMyDoc has collaborated with colleagues in the medical school, to develop a community-engaged learning activity for 2nd year medical students. Students attend a location in a global majority community, with the opportunity to provide blood pressure checks for attendees. After two pilot years, they have delivered 9 sessions involving 108 medical students in 2025/26.

## Discussion

### Positive takeaways

After 15 years of engagement with global majority communities, the key highlight is the positive impact of sustained contributions to health literacy. AskMyDoc has demonstrated that grassroots interventions require little financial cost but have maximal input. Key factors for effective community engagement include long standing trust between communities and healthcare teams, with equitable power dynamics and a collaborative approach (18). AskMyDoc has built relationships with local community leaders, to ensure maximal community engagement, and legitimacy of the activities. They have also ensured close collaboration with national charities and healthcare bodies. This enables a coordinated approach to breaking down barriers to healthcare. The work has been recognised, including being shortlisted for the Health Services Journal 2024 Awards-EDI award.

There have also been opportunities to develop scholarly work, based on the activities. This included presenting a workshop on ethnic minority health at the 2018 Shared Health Foundation Inclusion Health Conference, as well as a workshop for medical students at the 2020 University of Manchester GP Society Conference. They were also invited to deliver a training course on ethnic minority health to GPs in 2019, at a GP Hot Topics Course.

### Challenges

AskMyDoc’s work has not been without challenges. A key issue, also faced by other similar charities, is creating a pool of volunteers that can deliver the workshops. Restrictions for doctor volunteers include rota commitments, the lack of remuneration and changing roles every few months. This has resulted in Trustees needing to step into activities, which is difficult due to their busy schedules, or risk an event not happening. To ensure sustainability, there needs to be flexibility with commitment to the community engagement, and fair remuneration to undertake the work. Remuneration has been achieved through the work with the university, as well as external funding streams, to ensure that volunteers are remunerated at a competitive rate for their sessional time. A sustainable approach may be to link the work of resident doctor volunteers to their portfolios and learning needs, to enable them to use their study time to help support activities. This approach would require engagement with Deaneries and Royal Colleges, difficult to undertake with the current small team.

Another challenge has been relying on Trustees to cover the administrative work, in addition to their other roles and commitments. During covid-19, Trustees spent many hours a week to edit and produce videos. They also had to spend time to liaise with AskMyDoc Champions, and to ensure external verification of the videos in different languages. AskMyDoc Trustees recognised the risk of burnout with this approach and made a decision to look for administrative support. AskMyDoc has recently employed an administrator, to coordinate their activities. This has enabled smoother operation and delivery of work, particularly to support website development and outreach activities. However, funds are limited to expand the paid team, and the infrastructure is not in place to ensure permanent positions.

### Future perspectives

The AskMyDoc model has been successfully delivered locally and nationally. In 2021, a new branch was created in Slough, with the purpose of delivering health awareness workshops to global majority patients there. The innovative work there, organised by Dr. Hussain Mahmood, has included awareness activities on nitrogen oxide misuse. The hope is to spread the vision of engagement, education and empowerment to other global majority communities in the UK. This is through a sustainable model of grass-roots engagement, with equitable remuneration and active partnership with local global majority communities.

## Data Availability

The original contributions presented in the study are included in the article/supplementary material, further inquiries can be directed to the corresponding author.
